# Implementation fidelity of a transition program for adolescents with congenital heart disease: the STEPSTONES project

**DOI:** 10.1186/s12913-022-07549-7

**Published:** 2022-02-05

**Authors:** Markus Saarijärvi, Lars Wallin, Philip Moons, Hanna Gyllensten, Ewa-Lena Bratt

**Affiliations:** 1 Institute of Health and Care Sciences, University of Gothenburg, Gothenburg, Sweden; 2 KU Leuven, Department of Public Health and Primary Care, Leuven, Belgium; 3 School of Health and Welfare, Dalarna University, Falun, Sweden; 4 University of Gothenburg Centre for Person-Centered Care (GPCC), Sahlgrenska Academy, University of Gothenburg, Gothenburg, Sweden; 5 Department of Paediatrics and Child Health, University of Cape Town, Cape Town, South Africa; 6 Department of Pediatric Cardiology, The Queen Silvia Children’s Hospital, Gothenburg, Sweden

**Keywords:** Adolescent, Heart defect; congenital, Chronic disease, Implementation fidelity, Mixed methods, Process evaluation, Randomized controlled trial, Transition of care

## Abstract

**Background:**

Although transition programs have been evaluated for adolescents with chronic conditions, these have rarely involved process evaluations. Indeed, outcomes of complex interventions are dependent on how the intervention is implemented in practice and evaluations of implementation process are therefore pivotal. The aim of this study was to evaluate the extent to which a transition program for adolescents with congenital heart disease was delivered as intended. Research questions were 1) to what level of fidelity was the program delivered? and 2) what potential moderating factors affected the delivery of the program and overall fidelity?

**Methods:**

A mixed methods design was used, where a process evaluation was embedded in the STEPSTONES randomized controlled trial in Sweden. The implementation fidelity framework by Carrol (2007) and Hasson (2010) was used to design, collect and analyze data. Quantitative data consisted of intervention records on adherence and were analyzed with descriptive statistics. Qualitative data on moderators affecting fidelity were collected through interviews, log-books and focus group interviews with healthcare professionals implementing the intervention and participatory observations of the implementation process. Data were analyzed with deductive content analysis. Triangulation was used to integrate quantitative and qualitative data within the fidelity framework.

**Results:**

Six out of eight components of the transition program were delivered to an extent that adhered to the program theory or achieved a high level of fidelity. However, components involving peer support had a low attendance by the participating sample (32.2%), and the joint transfer meeting was challenging to implement, despite achieving high adherence. Moderators affecting the implementation process were the adolescent’s and healthcare professional’s engagement in the intervention, contextual factors and a lack of standard operating procedures for all components in the program.

**Conclusion:**

Barriers and facilitators for a future implementation of transition programs have been illuminated in this study. The use of an implementation fidelity framework in the process evaluation proved successful in providing a comprehensive evaluation of factors affecting the implementation process. However, implementation fidelity must be considered in relation to adaptations to the local and personal prerequisites in order to create interventions that can achieve fit.

**Supplementary Information:**

The online version contains supplementary material available at 10.1186/s12913-022-07549-7.

## Background

To facilitate the transition to adulthood and transfer to adult care for adolescents with chronic conditions (CC) transition programs have been developed and evaluated through randomized controlled trials (RCT) [1]. Transition programs are complex interventions. Consequently they target multiple organizational levels (i.e. pediatric and adult care), consist of several interacting components and require behavior change by those delivering and receiving the intervention. In addition, the causal pathways leading to outcomes are hidden and affected by several factors in the implementation process and surrounding context [2, 3]. However, the implementation processes of transition programs have been scarcely evaluated [3]. Such evaluations are considered process evaluations, where the implementation process, change mechanisms and contextual impact are explored in order to understand how and why outcomes are created. Implementation can both describe the process of translating evidence-based knowledge into clinical practice and implementation within an effectiveness evaluation, such as an RCT [2]. If not assessing how complex interventions are delivered and implemented, perfect implementation is assumed, which is rarely or never the case [4].

Within process evaluations, implementation fidelity is investigated [5–7]. This concept refers to the degree to which an intervention is delivered according to protocol [5]. The relationship between fidelity and trial outcomes are well known [5]. However, evaluations of fidelity have been debated, as interventions aiming to be tailored and adapted to the local context have proven to be more successful than interventions strictly adopted from protocol [8]. Thus, in addition to assessing fidelity, it is pivotal for evaluations of complex interventions to include investigation of the implementation process, exploring how the intervention was delivered in order to understand whether a successful trial was due to successful implementation, or if a failed trial was due to poor implementation [4].

This study is part of the STEPSTONES (Swedish Transition Effects Project Supporting Teenagers with chrONic mEdical conditionS) project which aims to design, evaluate and implement a transition program for adolescents with congenital heart disease (CHD) in Sweden [9, 10]. The aim of this study therefore was to evaluate to what extent the transition program was delivered as intended. Research questions related to the aim were 1) to what level of fidelity was the program delivered? and 2) what potential moderating factors affected the delivery of the program and overall fidelity?

## Methods

A mixed methods design was used in this process evaluation study in order to provide a comprehensive evaluation of factors affecting implementation fidelity. The embedded design was chosen, as qualitative and quantitative data on implementation were embedded in the STEPSTONES RCT [11]. A protocol describing the full extent of the process evaluation has been previously published [10]. The Good Reporting of A Mixed Methods Study (GRAMMS), along with the Consolidated criteria for reporting qualitative research (COREQ) checklist was used in the design and reporting of the manuscript.

### The STEPSTONES transition program

**Fig. 1:**
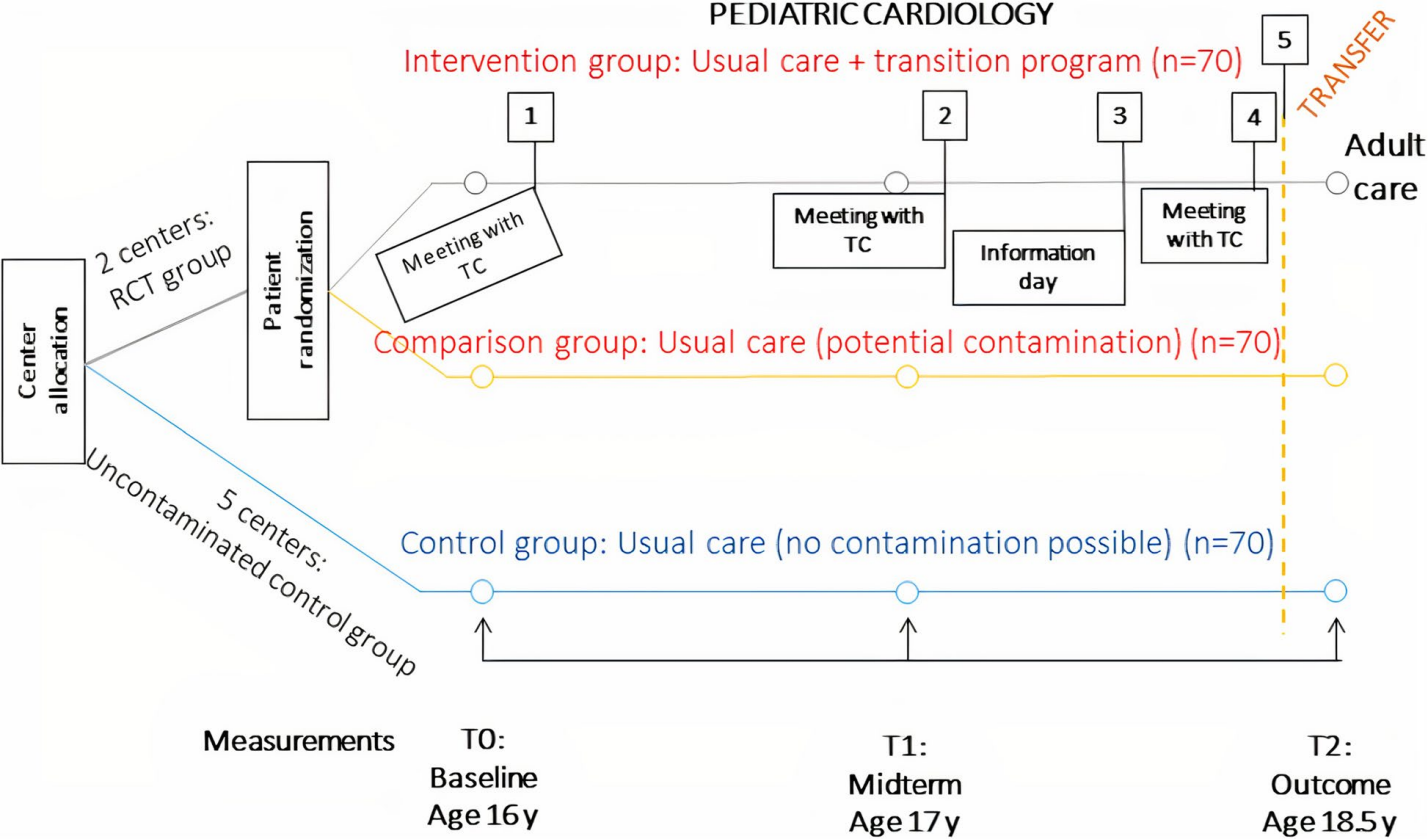
Flowchart of RCT design of the STEPSTONES project

The transition program was developed through intervention mapping to ensure an evidence-based and theoretically rigorous intervention in line with the stakeholders’ needs [12, 13]. The RCT was carried out in seven outpatient pediatric cardiology units in Sweden between 2016 and 2021. The primary outcome was patient empowerment [14]. Empowerment is an asset related to the individual’s capacity to manage their health and care, involving control and influence over their condition [15]. Based on the primary outcome, a sample size of 210 patients were targeted in a three-armed design (Fig. 1) in order to handle potential contamination of the intervention to usual care [9]. As the intervention was delivered at two of the seven centers, only participants and healthcare professionals (HCPs) enrolled in these two centers were part of the process evaluation.

**Fig. 2:**
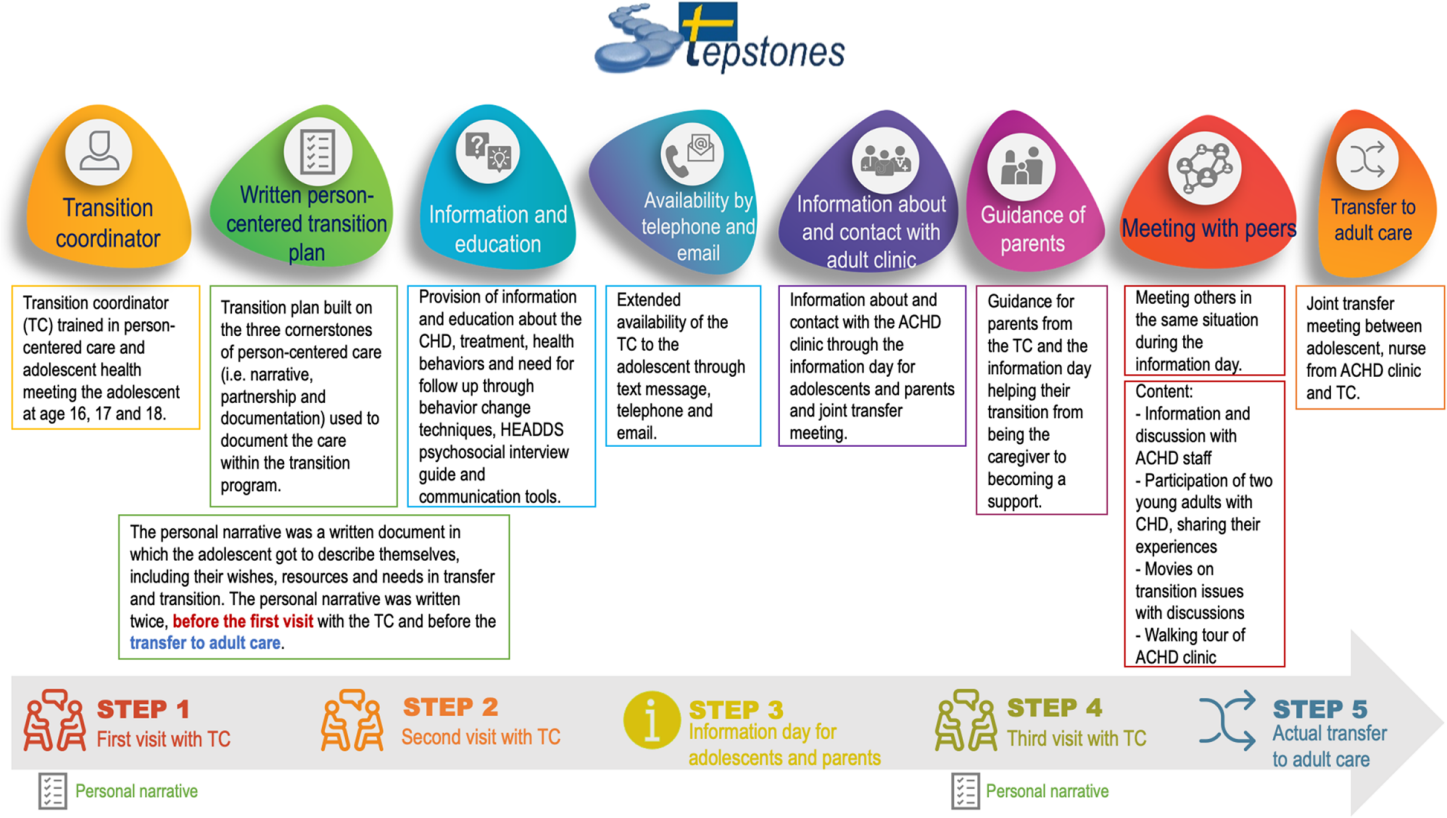
Components and implementation steps of the STEPSTONES transition program. HEADDS (Home Education Activities Depression Drugs Sexuality). TC = Transition coordinator

The transition program consisted of eight components delivered through five implementation steps (Fig. 2). However, the components were not strictly delivered from step 1–5, but aimed to be tailored to the adolescents’ needs. Person-centered care (PCC) along with knowledge and skills in adolescent health were the theoretical foundations on which the intervention was built [9, 10]. Furthermore, behavior change techniques to achieve patient empowerment were applied based on the needs assessment of the target population from previous studies [13, 16–19].

### Methodological framework

**Fig. 3:**
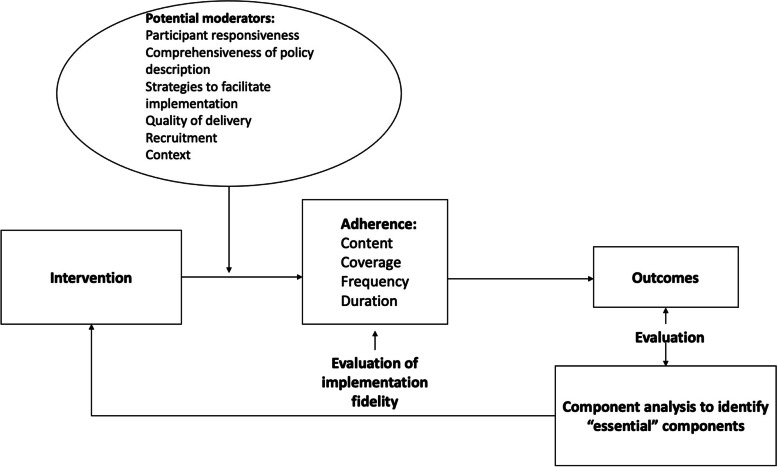
Implementation fidelity framework by Carrol et al. (2007), further developed by Hasson (2010)

The implementation fidelity framework by Carrol et al. (2007), further developed by Hasson (2010), [5, 6] was used to guide qualitative and quantitative data collection, analysis and integration (Fig. 3).

The evaluation of implementation fidelity is focused on the concept of *adherence*, which is defined as whether ‘a program service or intervention is being delivered as it was designed or written’ [20]. Adherence includes the subcategories’ content, frequency, duration and coverage, which refers to the dose of the intervention received by participants [5, 21]. Table 1 provides an overview of the constructs of implementation fidelity with related data sources in the present study.

**Table 1:** Overview of constructs of implementation fidelity, potential moderators, and data sources investigating these

	Adherence				Potential moderator				
	Dose	Content	Frequency	Duration	Quality of delivery	Participant responsiveness	Facilitation strategies	Recruitment	Context
Quantitative data sources									
Transition plan	X	X	X	X	X				
Intervention implementation forms	X	X	X	X	X				
Qualitative data sources									
Paired interviews with transition coordinators		X			X	X	X		X
Log-books of transition coordinators		X			X	X	X	X	X
Focus group interview with ACHD nurses		X			X	X			X
Participatory observations		X			X	X	X		X

Fidelity is also proposed to be moderated by six factors [5, 6], five of which are investigated in this study and presented in Table 1. *Quality of delivery* is the manner in which the HCPs deliver the program in relation to the theoretical ideal and program content [21]. *Participant responsiveness* relates to how participants respond to or are engaged by an intervention. It refers to both the individuals receiving the intervention (i.e. adolescents and parents) and those delivering it (i.e. HCPs). *Facilitation strategies* are approaches used to enable implementation and can be the use of manuals, guidelines, and training, along with monitoring and feedback for those delivering the intervention [5]. In the present study, we expanded this concept by also investigating what strategies HCPs used to overcome obstacles in delivering the intervention. *Recruitment* focuses on procedures to approach and retain participants to the study, and finally, *context* refers to if and how the intervention was adapted to the local prerequisites. Context takes into account the social, organizational, and cultural events in the assessment of implementation [6] (Table 1). Comprehensiveness of policy description was not investigated as it was beyond the scope of this study.

### Study sample and data collection

#### Quantitative data

Adolescents randomized to the transition program at the two intervention centers (*n* = 67) were sampled and inclusion criteria followed those of the RCT [9, 10]. Quantitative data were collected longitudinally in parallel with the effectiveness evaluation. Two primary data sources were used: 1) transition plans (i.e. intervention records) of the included patients which provided data on adherence, dose and quality of delivery (Supplementary file 1), and 2) intervention implementation forms, which provided detailed data on content and dose for each individual encounter (i.e. face-to-face, telephone, text message) in the transition program (Table 1) (Supplementary file 2). Adverse event reports were also included to ensure patient safety [9, 10]. To ensure validity of data they were cross-checked with the enrolment form of the RCT before analysis.

#### Qualitative data

A multilevel nested sampling was employed where the qualitative sample was nested within the quantitative sample of the intervention study [22]. This sample included participating adolescents and parents in the transition program along with HCPs. HCPs included the transition coordinators (TC) responsible for delivering the intervention, adult and pediatric HCPs (i.e. nurses, physicians, counselors and physiotherapists) and two patient representatives involved during the information day for participating adolescents and parents. Four qualitative data sources were developed for this study and evaluated implementation and potential moderating factors (Table 1) and were collected by the first author (MS), which was only involved in the process evaluation of the RCT.

##### Paired interviews with TCs

TCs (*n* = 2) working at each intervention center were interviewed together four times throughout the intervention study. Semi-structured interviews (Supplementary file 3) were carried out in 6–12-month intervals, where they were asked to reflect on their experiences of working as TCs in the transition program along with barriers and facilitators in program implementation. Paired interviews allowed for their joint perspectives to be explored, and deepened the understanding of similarities and differences in these experiences [23]. The interviews ranged between 63 and 68 min (m = 66) and were audio-recorded and transcribed verbatim by the first author (MS).

##### Log-books of TCs

Throughout the intervention study, the TCs were asked to keep log-books where they reflected on the experiences of working in the transition program along with barriers and facilitators to program implementation. These log-books allowed for individual reflection and for the TC to write about aspects of the transition program that might be lost in the interviews due to recall bias.

##### Participatory observations

To explore how the transition program was implemented in practice, participatory observations of the outpatient visits with TCs (*n* = 17) and information day for adolescents and parents (*n* = 3) were carried out longitudinally. In addition, informal interviews were carried out with the TCs if needed to verify or discuss the observation. In the observations of the outpatient meetings, sampling strived for variation [24] in terms of intervention center, visit observed (i.e. visit one, two or three), adolescents’ sex, and disease complexity of the CHD. During the observations, the observer (MS) did not interact with the participants if not approached. Participants in the observations were asked beforehand if they consented to the observation, with the option to decline. No participants declined observation. At the time of observation, the observer introduced himself and received written and oral informed consent from the participants. The same procedures were followed for the observations of the information day for participating adolescents and parents, except that additional written informed consent was not retrieved from the participants during these meetings. The field notes were handwritten and transcribed in close connection to the observations to ensure trustworthiness [25].

##### Focus group interview with adult congenital heart disease (ACHD) nurses

A focus group investigating the experiences of the transition program was performed with nurses (*n* = 4) working at the ACHD clinics at both intervention centers. The focus group was semi-structured (Supplementary file 4) and lasted 55 min and was held in December 2020 through the video conferencing software Zoom™ as the intervention centers were located in different cities and due to the Covid-19 pandemic [26]. The focus group was audio-recorded and transcribed verbatim by the first author (MS).

### Data analysis

#### Quantitative data

To estimate the level of adherence, data on dose, content, frequency and duration for the components and implementation steps were analyzed together, corresponding to the components of the transition program. Data were analyzed using descriptive statistics in the Statistical Package for Social Sciences (SPSS) v.24 and presented in absolute numbers and proportions. To estimate the level of fidelity, we created four categories based on each quartile of the percentages from 0 to 100 (i.e. low, somewhat low, somewhat high and high).

#### Qualitative data

Qualitative data on potential moderating factors to implementation fidelity were analyzed using deductive content analysis according to Kyngäs and Kaakinen (2020), using the five domains of implementation fidelity moderators as a coding matrix [27]. The analyses were performed by two authors (MS & ELB) in NVivo v.12 and discussed among the co-authors to ensure trustworthiness. First, qualitative data from the four sources were read through to get a grasp of the whole. Second, coding of meaningful units of text related to the aim was performed and sorted into the five domains of the fidelity framework. Third, codes were compared and merged together within the framework’s domains.

#### Mixed method integration

Qualitative and quantitative data were collected and analyzed separately. However, to increase the understanding of implementation fidelity and moderating factors, method triangulation was used to integrate the findings [11, 28]. Triangulation served the purpose of validating findings between different sources (e.g. using findings from participatory observations to validate findings from interviews) and to emphasize different aspects of the implementation process. In addition, data from qualitative sources were used to explain and understand findings from the quantitative sources and give answers to potential pitfalls in implementation [4]. Qualitative and quantitative data have therefore been presented together in the results according to the conceptual framework of implementation fidelity.

## Results

The characteristics of the participating sample in the intervention group (i.e. process evaluation sample) and participants in the RCT is described in Table 2. From the 67 adolescents randomized to the intervention group, eight dropped out before the first implementation step, meaning only the remaining 59 adolescents were included in the analysis and results.

**Table 2:** Demographic characteristics of process evaluation sample in relation to RCT-sample

Characteristics	Process evaluation sample *n* = 59 (%)	RCT sample *n* = 128 (%)
Female sex	26 (44.1)	59 (45.7)
*CHD complexity*^*a*^		
Mild	9 (15.3)	12 (9.4)
Moderate	31 (52.5)	81 (63.3)
Severe	19 (32.2)	35 (27.3)
*Comorbidities*	18 (30.5)	28 (21.9)
*Centre*		
Centre 1	44 (74.6)	91 (71.1)
Centre 2	15 (25.4)	37 (28.9)

^a^CHD complexity categorized according to 2018 AHA/ACC Guideline for Management of Adults with Congenital Heart Disease

### Adherence and moderating factors on implementation fidelity

Table 3 describes adherence to the components of the transition program along with the categories of fidelity. Below, adherence is presented together with quality of delivery.

**Table 3:**
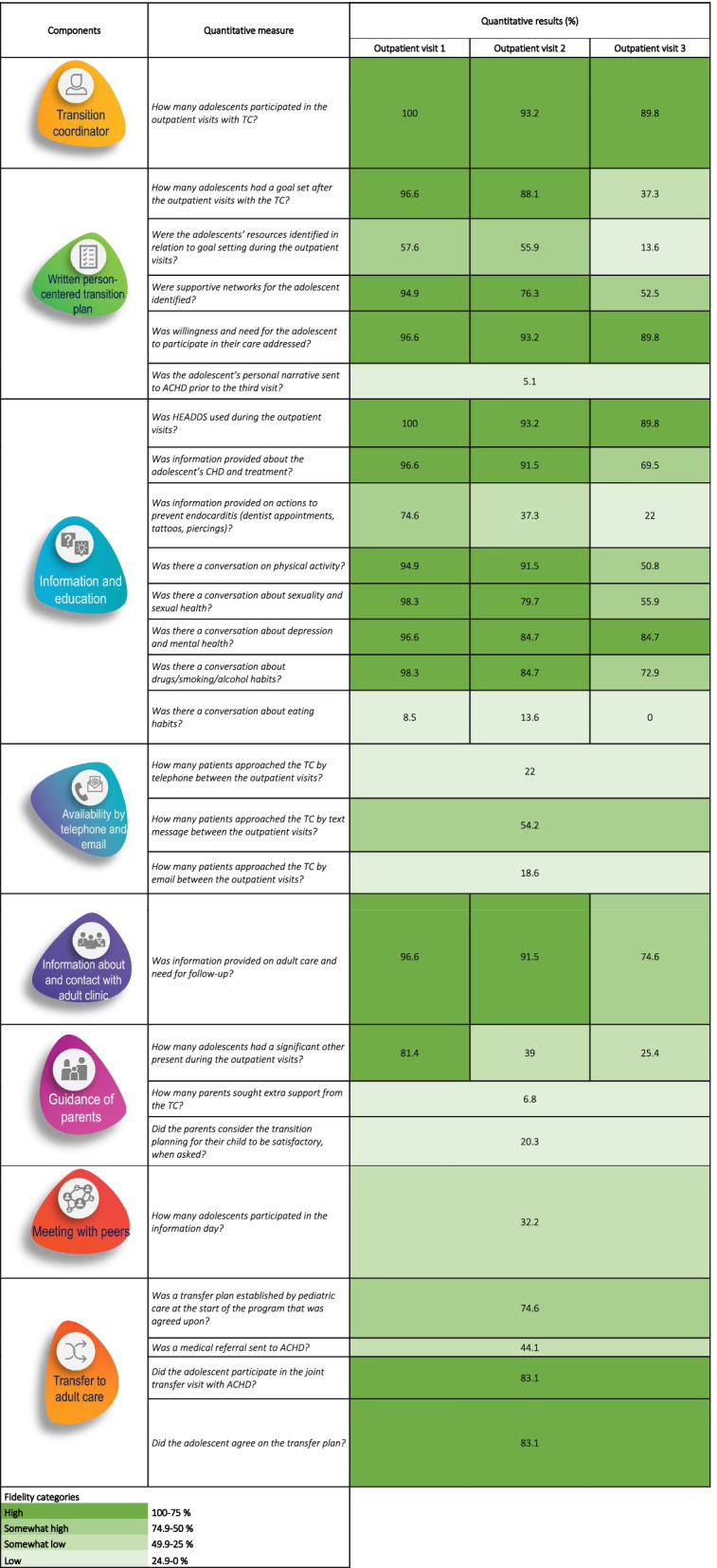
Fidelity of the components and implementation steps of the transition program

#### Quality of delivery and content delivered

Quality of delivery investigated the manner in which the intervention components were delivered and is presented according to the eight components of the transition program.

##### Transition coordinator

There was high attendance by the adolescents to the three outpatient visits with the TC (Table 3). According to the observations, the TCs followed the SOPs to a high extent, yet stayed true to the cornerstones of PCC. Moreover, during the interviews, the TCs expressed enthusiasm for the intervention and for being able to meet the adolescents in this format, which served as a motivation for the TC.

##### Written person-centered transition plan

The implementation of PCC components varied. Goal setting, identification of supportive networks and participation in care were carried out to a high extent during the first and second outpatient visit (Table 3). The transition plan was jointly documented by the adolescent and TC during the outpatient visits, a central part of the adolescent’s participation in the plan being to formulate, write and carry out goals for their health in transfer and transition. In all observations of the outpatient visits with the TC (*n* = 17), the adolescent along with the TC formulated goals. However, the TC sometimes needed to guide the adolescent in goal setting by using the adolescent’s personal narrative as a facilitator. The TCs expressed that goal setting was difficult for adolescents who were perceived as disengaged or who had few symptoms of their CHD. Throughout the course of the trial, the TCs found it easier to support adolescents in goal setting as they found strategies to deal with this (Table 4).

**Table 4:** Illustration of domains of the fidelity framework with quotes and excerpts from field notes

Domain	Sub-domain	Quote
Quality of delivery and content delivered	*Written person-centered transition plan*	*“I do have a large number with goals that aren’t at all related to their heart defect but that they feel very motivated about, so I try to find what they want to work on because I think it isn’t a question of...of us wanting to achieve empowerment so maybe it doesn’t matter if they manage to learn about their heart condition inside out or if they manage to get a pass in maths. It might have more to do with feeling capable and that I’ve succeeded at something – whatever it is. So trying to find something we can improve so they can feel they are developing in it.”* - Paired interview no. 2 with Transition Coordinators
	*Information and education about the condition and information and contact with the adult clinic*	*“They’re still discussing how the heart defect affects social aspects. The youngster says her classmates don’t know about her heart defect. The reason is she wants to keep her condition to herself and is scared that things will change in class if they find out about it. She then says one person in the class knows about her heart defect – “It depends on how close you are to that person”, she says. The TC confirms that the youth can decide for herself whether to tell the others in her class about her heart defect or to keep it to herself.”* - Field note, observation 2, visit 1, center 2
		*“The TC starts explaining the anatomical heart model and a colorful picture of the heart. She first explains normal physiology, before going on to describe by drawing what kind of heart defect he had from birth. And then the TC feeds back that this is complex knowledge and that he doesn’t have to understand everything now and it will come gradually. Then she explains what operations have been done. The youth says he didn’t actually know a number of things the TC is explaining. The TC then takes out more pictures of what the heart looks like now, after the operation. The TC keeps reiterating that this is very difficult to understand and that you have take it step by step. She asks if his head is spinning – a bit, says the youth. The TC then asks him what he thinks is the best way to learn this. Bit by bit, replies the youth. The TC also explains that it depends on how much you want to learn. They both conclude that this is the goal – to learn about the heart defect from the beginning. The TC now asks how he thinks he wants to learn. He wants to read up on it to learn. The TC explains she has prepared educational materials for other patients and emphasizes that he doesn’t need to know everything by next time – it isn’t a test.”* Field note, observation 9, visit 1, center 2
Participant responsiveness	*Moderators of adolescents’ responsiveness*	*“There was a girl I met on the first visit... I can’t remember when she was born but she was suffering badly with her mental health from the start and we weren’t focusing on the transition or heart defect at all – we couldn’t approach that. So the conversation wasn’t at all as we had planned. But when she came for the next visit, even though the last conversation had been of a different character it meant we had built some kind of trust – I was familiar with her situation and had a completely different starting point from which to approach these questions that I had been meaning to ask in our first meeting”* - Paired interview no: 4 with Transition Coordinators
		*“The TC goes on to ask the youth what she knows about her heart defect. This makes the youth a bit nervous. It feels like the conversation is starting to take a turn here – the youth isn’t as responsive but just gives shorter and shorter answers. Then the youth says to herself: I should know this. The TC replies: it isn’t a test.”* - Excerpt from field note from observation no: 6, first visit in the transition program at Center 2.
		*“Because I have some I’d like to meet every three months to work more intensively on certain things... medication management, for example, quite often it can be a year...that’s kind of too long! And for some it is...”* - Paired interview no: 3 with Transition Coordinators
	*Moderators of the TCs’ responsiveness*	*“We have quite a few with ADHD … And how to reach them as well as everything going on in their heads. How they say their heads are boiling over and it’s really hard to take in what it should be like when their heads are just fizzing and they say that one of their mates usually smokes some weed and then they get much calmer... “Yeah, okay, how do you reason with that? So it would have been really exciting to speak to someone who meets these youngsters more often and simply understand what the scenarios are and stuff. Because these are things you get stumped by. Not being taken aback and being able to carry on and have a good conversation, because these are things they can’t really tell their parents either, even if they have a good relationship with them. But it’s exciting... and a bit like, ‘oh’”* - Paired interview no: 2 with Transition Coordinators
		*“I think it’s so much fun to put them in control – they’re not used to it. And I think it’s especially cool in the second meeting when they arrive and have that attitude and start talking, and it feels like they... that you can give them that – that they are allowed to speak up about what they want and we can give them that. I think that’s cool, and it feels like that’s not always the case in other care I’ve administered, but it’s been: this is what you need and we’ve decided what you need. It’s fun to turn it around.”* - Paired interview no: 2 with Transition Coordinators
	*ACHD nurses’ responsiveness*	*“Nurse 2 from Center 1: Yes, just like you say – sometimes, you’d just like to have a template for what issues you’re expected to raise, because we just wing it really – and then there are some things, like with studies, and when you’re supposed to be here, how you’re feeling... I mean the usual things, you deal with those anyway. But if there is something specific – then it would have been good...* *Nurse 1 from Center 1 = Yes* *Nurse 2 from Center 2 = Yes, I agree with that”* - Excerpt from focus group with ACHD nurses
Facilitation strategies	*HEADDS and communication tools – facilitating the partnership*	*“The TC starts the next speech bubble, which is sexuality and contraception. She doesn’t say the word, just points at the bubble. The youth says she hasn’t thought about it much before. The TC tells this youth she has to get in touch with a gynecologist if she wants the pill due to increased risk of blood clots with this specific heart defect. Then the TC carries on talking about pregnancy – that it’s not an obstacle for her but that she would probably need more monitoring during a pregnancy. The youths says she has thought about this a bit and didn’t think she could have children and wonders if there’s a risk her heart defect is hereditary. The TC explains it isn’t and that she can get pregnant. The youth seems to be happy about that but explains she had thought about adopting anyway as there are so many children in the world who need a parent. The TC gives her affirmation for this and says she is free to choose what she wants to do.”* - Excerpt from field note from observation no: 2, first visit in the transition program at Center 2.
Context	*The impact of social context on implementation*	*“If you’re not allowed that freedom to think about what you want to do... they describe quite strict restrictions on leisure activities and choice of profession and so on... then you don’t reflect on your limitations either if someone else is in the driving seat. You’re not even allowed to reflect on it. It’s not until you face a problem that you reflect on the solution, if someone else is always in the driving seat.”* - Informal interview with TC after participatory observation no: 6 in Center 2.

The most difficult part of goal setting was not formulating a goal, but finding the resources the adolescent felt they needed in order to reach their goal (Table 3). From the interviews with TCs it was evident this was a recurring challenge, and the observations confirmed this as a barrier, with both the adolescents and TCs struggling to grasp these aspects. As a result, goal setting was not always performed, as the TCs did not want the adolescents to lose interest or disengage. Additionally, goal setting was not always performed during the final outpatient visit because many adolescents wanted to start in adult care on a ‘clean slate’ (Table 3).

In their interviews and logbooks the TCs said that the written personal narrative helped them become acquainted with the adolescents. However, personal narrative was implemented to a low degree before the transfer to adult care, with 5.1% of adolescents completing this narrative. Potential reasons were that the adolescent forgot to write and send in the narrative before the visit, that the adolescents felt it was awkward to write a personal narrative to someone they did not know, and that the ACHD clinic did not have any way of storing the transition plan as an electronical medical file.

##### Information and education about the condition and information and contact with adult clinic

The psychosocial interview guide HEADDS was used to a high extent in the first visit, but to a lesser extent during the second and third visit (Table 3). The TCs stated that performing HEADDS during the second and third visit was sometimes repetitive. But as time went by, the TCs built on their experience and found ways to facilitate the use of HEADDS in the visits. The observations showed that the visits began with a familiarization phase between the TC and adolescent through the use of HEADDS. By covering all domains in HEADDS, the TC made the adolescents feel relaxed and talkative, which facilitated the establishment of a partnership. Hence, when the TC brought up challenging issues, such as the adolescents’ CHD and its impact on daily life, most of the adolescents felt comfortable talking about these issues (Table 4).

From the quantitative data and observations it was evident the adolescents generally received tailored information, with the first visit being more informative than the latter in terms of level of fidelity (Table 3). In order to achieve tailored information and learning, behavior change techniques such as active learning, goal setting, modeling and verbal persuasion were used to a high extent during the outpatient visits and information day (Table 4, example of active learning).

##### Availability by telephone and email

In between the visits, the amount of contact between the adolescent and TC was low. Of the available mediums, text message was most commonly used (Table 3). The observations showed that the TC informed the adolescents about the option to contact them between the visits if they had questions or concerns. The logbooks and interviews also revealed that the TCs perceived this as an extra important feature of the intervention for some adolescents, while for some it was unnecessary.

##### Guidance for parents

The implementation of this component varied. A high proportion of adolescents had a parent, caregiver or sibling present during the first visit (81.4%) but a low proportion of parents sought extra support from the TC (6.8%). The observations showed that parents were present during the first 10–15 min of the outpatient visits. During this time, the TC opened up for discussion on the transition program, addressing the shift in responsibility from the parent to the adolescent.

##### Meeting with peers during the information day for adolescents and their parents

During the information day, information on the transfer to adult care and the opportunity to meet others in the same situation was delivered to the participating adolescents and parents by HCPs from adult care (i.e. nurses, physicians, counselors and physiotherapists), the TC, and two young adults from the patient organization of CHD. Three information days were held throughout the course of the trial, two in center 1 and one in center 2, where a total of 32.2% of the adolescents in the transition program participated.

##### Transfer to adult care

A high proportion of adolescents participated in the transfer meeting and were satisfied with the arrangements made for the transfer. However, medical referral was sent to a low extent prior to this meeting (Table 3). The qualitative data showed that a potential explanation for this was that the transfer meeting in the transition program was not always synchronized with the medical transfer in usual care.

#### Participant responsiveness

##### Moderators of adolescents’ responsiveness

Four factors were identified from the qualitative data that moderated the adolescents’ responsiveness to the transition program. The first factor was their level of maturity. The TCs struggled to engage adolescents who were perceived as immature when implementing components such as discussing health behaviors, goal setting and initiating conversations about the transfer and transition. In addition, recruitment in the later stages of the RCT involved patients that were 15 years old at recruitment. The TCs experienced that these patients were harder to engage in relation to their age. The second factor was mental health issues along with neuropsychiatric variations. As the intervention involved discussions on aspects related to everyday life, these issues were considered a barrier towards engagement (Table 4). The third factor was disease complexity and the perceived need of the transition program. The TCs found it challenging to engage adolescents with a mild CHD, as it had little impact on their daily life. In contrast, patients with a complex CHD generally had more symptoms and therefore a greater need of the intervention. However, there were adolescents with mild CHD who expressed worries regarding their CHD and transfer to adult care and therefore, for some individuals, the perceived need of the intervention was a greater influence. The fourth factor, identified by the observations, was the adolescent’s willingness to talk and engage in their CHD. When the TC directed the conversation to their CHD or the transfer to adult care, these adolescents became disengaged (Table 4). Additionally, the time between the implementation steps (i.e. outpatient visits) was sometimes considered a barrier, as 1 year between the visits in the transition program could be too long for some adolescents to be comfortable and find meaningful output (Table 4).

##### Moderators of the TCs responsiveness

Three factors were identified. First, it was essential for the TC to become accustomed to the new role. Early in the implementation of the transition program, the TCs were still finding their professional role in terms of boundaries, responsibilities and managing challenging patient situations. However, by building experience and sharing these with each other, the TCs gained coping strategies. Second, lack of knowledge sometimes gave a feeling of inadequacy. This was related to meeting patients with mental health issues and neuropsychiatric variations, for whom the TCs felt their training and role was sometimes insufficient in meeting the patients’ needs (Table 4). The third factor was the TCs, ability to shift from working strictly to the intervention protocols to tailoring the intervention to the adolescent. This entailed letting the adolescent control the conversation and find aspects important for them to work with, which had positive effects on the TCs’ experiences (Table 4).

##### ACHD nurses’ responsiveness

The ACHD nurses perceived the transition program as an important intervention. They valued being able to participate during the information day for adolescents and parents, as it gave them the opportunity to bring up important issues in adult care and adult life before the transfer. The transition program also gave the nurses assurance that the adolescents were prepared and informed. Nevertheless, there were challenges when it came to changing the current way of working and a need to safeguard the pre-existing transfer protocol. Center 2 had a transfer meeting between the physicians from pediatric and adult care which the ACHD nurses felt conflicted with the transfer meeting in the transition program, even though they felt the content of the two meetings differed. In addition, several shortcomings were identified in the joint transfer meeting between the TCs and nurses. Generally, the nurses felt unprepared for the meeting, lacking a structure, purpose and role. The observations showed that the nurses often expected the TC to lead the conversation. Moreover, the nurses were unprepared for which patient they were going to meet because it was not part of the standard operation procedures (SOP) for the TC to report any patient information prior to the meeting. To facilitate future implementation of this component the nurses requested more information or a protocol on which topics were to be covered (Table 4).

##### Moderators of responsiveness during the information day

Four moderators of the participants’ engagement during these information days were identified. First, the use of movies enhanced the group discussions. The movies dealt with experiences of transfer and transition, Q&A of common medical issues, and social, vocational and financial issues when living with a CC. The movies thus served as icebreakers and facilitated the discussion by making the adolescents comfortable in communicating with the HCPs. Second, the use of patient representatives created an informal atmosphere. The representatives candidly shared their experiences and acted as conversation starters. The third moderator was the HCPs’ level of comfort in sharing power and meeting the participants on their terms. Two examples were identified. In example one, the HCPs tailored the information to the adolescents’ needs, which facilitated the discussion. In example two, the information was medically focused with use of complex terminology. This resulted in a group of disengaged adolescents. Fourth, the use of separate group discussions facilitated a safe space. For parents, this meant they could discuss worries and fears regarding the transfer and transition with parents in the same situation. For the adolescents, they could discuss sensitive topics (e.g. sex, alcohol and substance use) without parents present.

#### Facilitation strategies

##### Standard operation procedures and protocols (SOP)

SOPs were developed to guide the TCs in their work. Part of the SOP was to perform a medical chart review and consult the physician before each visit with the adolescent in the transition program. These procedures gave the TCs important medical information that prepared them and gave an understanding of the adolescents’ and families’ history. They also ensured patient safety, as the TCs were prepared for which information they could provide the adolescent within their field of responsibility. However, there were limitations in the SOPs related to the transfer meeting where the TCs lacked structure.

##### HEADDS and communication tools –facilitating the partnership

The HEADDS tool was appreciated by the TC in getting to know the adolescent as it covered aspects related to their everyday life rather than focusing on the illness. HEADDS was also effective in engaging adolescents who had low responsiveness by initiating topics related to their CHD and care in a natural way from the adolescents’ point-of-view.

To help the adolescents communicate their needs during the outpatient visits, communication tools were used. First, a shared decision-making model was used with the adolescent and parents to discuss the shift of responsibility from the parent to the adolescent during the transition to adulthood. Second, a concept map containing topics related to the CHD, care and adolescent life was used during the visits. The TCs experienced this tool as effective for adolescents who were untalkative or had cognitive challenges. Observations identified that these tools facilitated a more equal conversation, as the adolescents could choose topics to discuss. In addition, discussion of sensitive subjects (e.g. sex, drugs and alcohol) were facilitated (Table 4). Third, a linear analog scale was used to assess the adolescent’s quality of life and health status. The TCs stated that this enabled screening for both problems and resources and for some adolescents this was a starting point to formulate goals.

##### Utilizing the team in difficult situations

The TCs experienced that meeting the adolescents sometimes revealed mental, social and family issues that were beyond the scope of their professional role. The TCs handled this by utilizing the expertise of the surrounding team at the pediatric clinic (i.e. counselors, psychologists and physicians). However, safeguarding the adolescent’s confidentiality was sometimes considered a barrier to utilizing the team as the TCs felt obliged to protect this confidentiality.

##### Tailoring the intervention to the adolescents’ circumstances

The final facilitation strategy was related to tailoring the components of the intervention to achieve fit. As several components were focused on information and education through PCC, the TC used several strategies to achieve implementation of these components, such as engaging the adolescents by starting from their point-of-view, being flexible towards the adolescents’ needs and using information technology (i.e. smartphone Health-application). Moreover, as the TCs grew more confident in their role, they used these strategies when meeting adolescents who were disengaged or had other difficulties in expressing their needs.

#### Recruitment

The recruitment for the RCT spanned from June 2016 – December 2018. Three barriers and five strategies to recruitment and retention were identified. First, lack of engagement from the adolescents was most common according to the TCs. Second, problems with the postal services resulted in missed information to presumptive participants. Third, difficulties in reaching the participants were prevalent (e.g. no phone number registered in the adolescents medical file, adolescents living with separated parents, or that the information in their medical file was insufficient to recruit).

Strategies were used to handle these barriers. First, offering an incentive (i.e., cinema ticket) was perceived as encouraging the participants to be more engaged and continue study participation. Second, in some cases, using the parents as gatekeepers and contacting them first proved effective. Third, calling and texting was convenient as the adolescents were used to this form of communication and responded more promptly. Fourth, study information was adapted to each individual’s needs after reading about the adolescent in the medical file. And fifth, coordinating recruitment and outpatient visits in the intervention with the medical check-ups was an effective way for retention, as the patient was already at the clinic.

#### Context

##### The impact of social context on implementation

Social context surrounding the adolescent was a factor that affected implementation. Here, parents were considered to be both facilitators and barriers. Both interviews with the TCs and observations confirmed that some parents had difficulties in letting their adolescent manage their own care. On the other hand, the TCs considered most parents as resources to their child in the transition program. The TCs also felt the transition program gave insight into conflicts between the adolescent and parent that had to be considered by the TC (Table 4).

##### The impact of organizational context on implementation

As the transition program was delivered in addition to usual care, the TCs had to coordinate this. Three challenges were identified. First, the TCs had challenges in reaching the physician and finding a time for consultation before the outpatient visits. The TCs described having to be flexible and adjusting their schedule according to the physician’s needs, which was more commonly the case in center two than center one. The second challenge was related to the physical context, where center two had a designated office for the TC but center one had difficulties in finding a private space for the visits due to shortage of rooms in the outpatient pediatric cardiology clinic. The third challenge concerned attitudes from HCPs in usual care towards the intervention. Right from the start of the trial, the TCs described the HCPs as being skeptical towards them in their role. As the TCs had to safeguard the intervention’s content to avoid possible contamination of the intervention to usual care, they felt that this affected the general attitude. However, throughout the course of the trial these attitudes gradually changed towards a more positive outlook on the intervention and TCs (Table 4).

## Discussion

Our results show that the components of the STEPSTONES transition program were delivered to a high extent in a manner that adhered to the program theory. In addition, important moderators specific to transition programs (e.g. level of maturity of adolescents, cooperation between adult and pediatric care) and complex interventions for people with CCs have been illuminated. From our findings we identified two components that the participants found particularly difficult to engage in and implement - the information day for adolescents and parents and the transfer meeting with adult care. Indeed, only a third of adolescents in the transition program participated during the information day. Potential reasons for this are that adolescents with a CC might feel uncomfortable in engaging with others in the same situation and spend excessive time thinking about their illness [29, 30]. Within patient empowerment, the ability to enable others is a core construct [14, 15]. However, empowerment entails the individual being seen as a capable person with the ability to make informed decisions concerning their health [31]. Arguably, choosing not to participate in an information day can be explained by the fact that some adolescents are comfortable in their illness identity and do not feel the need to engage with others to be empowered [32, 33]. Nevertheless, when designing components involving peer support in complex interventions, these results emphasize the importance of considering the diverse needs of people with CCs and working with PCC to tailor components to achieve fit.

It was difficult to achieve a high level of fidelity for the transfer meeting to adult care. Reasons identified were the lack of SOPs concerning the content of the meeting, that adult care did not work with PCC, and the difficulties integrating the transition program transfer meeting with the transfer routines in usual care. These findings highlight important barriers to overcome for future implementation of transition programs and raise the issue of how to achieve fit for complex interventions into the complex social systems which healthcare systems exemplify [34]. Although complex, the STEPSTONES transition program did not involve components targeting the organizational levels within pediatric and adult care [9, 10, 13]. Arguably, this could be an additional reason for the difficulties in implementing this component and implementation step of the program.

Although implementation challenges and moderators of fidelity have been identified in this study, it is worth asking whether high fidelity and its assessment is important in process evaluations of complex interventions. When comparing interventions implemented strictly according to protocol with interventions that were tailored to the local context and patient population, the tailored interventions proved to be more effective in producing change [8]. Furthermore, person-centered interventions promotes active patient participation and adaptation of the intervention to the person’s preferences in a partnership between the patient and HCP [35]. Are evaluations of implementation fidelity redundant in these types of complex interventions? Perhaps, as the value of assessing implementation fidelity is to fully describe which components of the intervention were delivered and what the potential pitfalls and barriers to implementation were [4–6]. However, low fidelity from quantitative assessments does not always indicate poor implementation or inadequacies in the delivery of the program; instead it may indicate a high level of tailoring to the contextual and individual circumstances. Carrol et al. (2007) and Hasson’s (2010) have therefore proposed moderating factors that were explored qualitatively and shown to be important in nuancing the quantitative findings. As process evaluations tend to be extensive in the amount of collected data [4], the use of frameworks can also aid in defining the research question.

Some methodological caveats must be raised when interpreting these findings. First, the process evaluation of the STEPSTONES project covered several aspects of the implementation, context and potential mechanisms of impact of the transition program [10]. Consequently, there is a risk that the process evaluation and researcher performing the evaluation affected the effectiveness evaluation by being present during several implementation steps and by performing interviews with HCPs responsible for implementing the intervention [4, 36]. However, the researcher performing the data collection was not part of the analysis and interpretation of findings from the effectiveness evaluation. Second, sampling procedures aimed to capture a broad variety of perspectives by observing different outpatient visits at different points in time. However, only 17 observations were performed across a total of 167 outpatient visits, so these observations cannot provide a complete picture of the implementation of the program in practice. Nevertheless, this limitation is always present in process evaluations and was handled by triangulating findings from the observations with data from interviews, logbooks and the quantitative sources to ensure trustworthiness and achieve a deeper understanding of the implementation process and the moderators [11]. Moreover, as the process evaluation was performed longitudinally alongside the RCT, this enhances the trustworthiness of our findings. Third, quantitative data consisted of routine monitoring data from the RCT which might impact validity of these data as they were collected by the TCs. Thus, data from these different sources (i.e. transition plans and intervention implementation forms) were cross-checked with each other before analysis to ensure validity. However, the use of routine monitoring data might introduce bias which is a potential limitation of this study. Finally, as the data collection and analysis were performed before knowing the outcomes of the effectiveness evaluation we were unable to analyze which components were essential to outcomes which is an essential part of process evaluations [5]. However, this can be considered a strength as it may avoid biased interpretation of the effectiveness evaluation [4].

## Conclusion

The STEPSTONES transition program was delivered in a way that adhered to the program theory. Identified moderators affecting the implementation process were both the adolescents’ and HCPs’ responsiveness, contextual factors in pediatric and adult care, and the lack of comprehensive SOPs for all the implementation steps of the program. As a result, barriers and facilitators for a future implementation of transition programs into usual care have been illuminated. The use of an implementation fidelity framework to design and perform a process evaluation study proved successful in providing a comprehensive evaluation of factors affecting the implementation process. However, implementation fidelity must be considered in balance with adaptations to the local and personal prerequisites to create interventions that can achieve fit.

## Supplementary Information

**Additional file 1.**

**Additional file 2.**

**Additional file 3.**

**Additional file 4.**

## Data Availability

All data are available from the authors upon request.

## Abbreviations

ACHD: Adult congenital heart disease
CHD: Congenital heart disease
CC: Chronic condition
COREQ: Consolidated criteria for reporting qualitative research
GRAMMS: Good Reporting of A Mixed Methods Study
HEADDS: Home, Education, Activities, Depressions, Drugs, Sexuality
HCP: Healthcare professionals
LAS: Linear analog scale
PCC: Person-centered care
RCT: Randomized controlled trial
SOP: Standard operation procedures
SPSS: Statistical package for social sciences
STEPSTONES: Swedish Transition Effects Projects Supporting Teenagers with Chronic Medical ConditionS
TC: Transition coordinator
