# Supplementary material for: Implementation fidelity of a transition program for adolescents with congenital heart disease: the STEPSTONES project
Source: BMC Health Serv Res. 2022 Feb 5;22:153. doi: 10.1186/s12913-022-07549-7 (PMC8817652; doi:10.1186/s12913-022-07549-7)
Supplement: Supplementary file 1 — Additional file 1. [file 12913_2022_7549_MOESM1_ESM.pdf]

Swedish Transition Effects Project Supporting Teenagers with chrONic mEdical conditionS

INTERVENTION IMPLEMENTATION FORM

Patient study ID: ________________________ **Date**: ____/_____/_____

Contact:

☐ Visit ☐Call ☐SMS ☐Email

Topics covered:

**Health condition (s):**<br>☐ConHD<br>☐Other health conditions:<br>__________________<br>☐Treatment	**Health behaviors:**<br>☐Physical activity<br>☐Drugs<br>☐Eating habits<br>☐Activities	**Empowerment:**<br>☐Self-management skills<br>☐Participation in care
**Endocarditis prevention:**<br>☐Dentist appointments<br>☐Tattoos<br>☐Piercings	**Sexuality:**<br>☐Contraceptives<br>☐Pregnancy<br>☐STD	**Education:**<br>☐School<br>☐Career planning
**Transfer to adult care:**<br>☐GUCH<br>☐Need for follow-up	**Support networks:**<br>☐Parents<br>☐Siblings<br>☐Friends<br>☐Partner	**Others:**<br>☐____________________<br>☐____________________<br>☐____________________

Recommendations:
