# Supplementary material for: Implementation fidelity of a transition program for adolescents with congenital heart disease: the STEPSTONES project
Source: BMC Health Serv Res. 2022 Feb 5;22:153. doi: 10.1186/s12913-022-07549-7 (PMC8817652; doi:10.1186/s12913-022-07549-7)
Supplement: Supplementary file 2 — Additional file 2. [file 12913_2022_7549_MOESM2_ESM.docx]

**GENERAL DETAILS**

| **Check list**  **This list should be checked at each visit and updated if necessary** | |
| --- | --- |
| Name |  |
| Personal identification number |  |
| Address |  |
| Mobile phone number |  |
| E-mail address |  |
| Permission to send e-mail |  |
| Permission to send text message |  |
| Permission to call |  |
| Has received information and granted confidentiality |  |
| **Guardian 1** | |
| Name |  |
| Address |  |
| Mobile phone number |  |
| E-mail address |  |
| Permission to send e-mail |  |
| Permission to send text message |  |
| Permission to call |  |
| Has received information and granted confidentiality |  |
| **Guardian 2** | |
| Name |  |
| Address |  |
| Mobile phone number |  |
| E-mail address |  |
| Permission to send e-mail |  |
| Permission to send text message |  |
| Permission to call |  |
| Has received information and granted confidentiality |  |
| **Other significant person** | |
| Relationship |  |
| Name |  |
| Address |  |
| Mobile phone number |  |
| E-mail address |  |
| **Special needs** | |
| Needs support at school  (if yes, what and how) |  |
| Impaired hearing |  |
| Disability  (if yes, what) |  |
| Needs an aid  (if yes, what) |  |
| Other significant diagnoses |  |
| Other |  |
| **Information about ongoing care** | |
| Paediatric cardiologist in charge |  |
| Other professions involved in their care (nurse, etc) |  |
| Name |  |
| Contact details |  |

| **Confirmed need of other specialist skills set at cardiac clinic** | | | | | | | | |  |
| --- | --- | --- | --- | --- | --- | --- | --- | --- | --- |
| Welfare officer (name/date) | | | | Physiotherapist (name/date) | | Psychologist (name/ date) | | |  |
| Other? If yes, state what profession (name and date) | | | | | | | | |  |
| **Team verdict** | | | | | | | | |  |
| Transfer plan drawn up and agreed according to the patient’s wishes: | | | | | | | Yes | No |  |
| Does the Guardian consider the transfer planning to be reasonable and satisfactory? | | | | | | |  |  |  |
| Aggravating circumstances: | | | | | | | | |  |
|  | | | | | | | | |  |
| Parents’ participation | | | | | | | | | |
| Attendance visit 1 (who) | Yes | | No | | Wishes/comments: | | | | |
|  | | | | | | | | | |
|  | | | | | | | | | |
| Attendance visit 1 (who) | Yes | | No | | Wishes/comments: | | | | |
|  | | | | | | | | | |
|  | | | | | | | | | |
| Attendance visit 1 (who) | Yes | | No | | Wishes/comments: | | | | |
|  | | | | | | | | | |
|  | | | | | | | | | |
| Transfer planning | | | | | | | | | |
| Participation youth day | Yes | | No | | Date | | | | |
| Referral to receiving unit sent | Yes | | No | | Name of receiving unit | | | | |
| Personal narrative written | Yes | | No | |  | | | | |
| Personal narrative sent with referral | Yes | | No | |  | | | | |
| Last visit to unit handing over | Date | | | | Family/friend accompanying on visit (state who) | | | | |
| Planning of first visit at the receiving unit  Date | | Contact at receiving unit (name) | | | | | | | |
|  |  | Doctor in charge of receiving unit (name) | | | | | | | |

**VISIT 1**

| **Preparations for visit 1** | |
| --- | --- |
| Update via medical journal |  |
| Send appointment request  (and parents’ brochure to parents) |  |
| Date and time agreed |  |
| Prepare documentation |  |
| Book meeting room |  |
| Ensure the material to be used is there (LAS scale, paper, pens, computer, plastic heart model etc) |  |
| Send text message reminder about visit |  |
| **To check with the paediatric cardiologist in charge**  **– aspects in relation to the person and the heart defect** | |
| Health status |  |
| Physical activity |  |
| Medication |  |
| Pregnancy |  |
| Contraceptives |  |
| Prognosis (short and long term) |  |
| Future need of intervention |  |
| Future need of medical checks and at which level of care |  |
| Advice regarding choice of education and profession |  |
| Advice regarding travel (to think about) |  |
| Driving licence |  |
| Endocarditis prophylaxis |  |
| Tattoos and piercings |  |

| **Patient reported health status/skills and resources – Visit 1** | | |  |
| --- | --- | --- | --- |
| **Date** | |  | |
| Health (1-10) LAS | |  | |
| LAS (1-10)  Based on person’s needs  Specify your assessment | |  | |
| LAS (1-10)  Based on person’s needs  Specify your assessment | |  | |
| **HEADSS** **– Visit 1** | | |  |
| **H**  **Home** |  | |  |
| **E**  **Education**  **Employment** |  | |  |
| **A**  **Activities** |  | |  |
| **D Drugs/alcohol/**  **smoking** |  | |  |
| **D**  **Depression**  **Mental health** |  | |  |
| **S**  **Sexuality** |  | |  |
| **Strengths** |  | |  |
| **Participation** |  | |  |
| **Knows about their heart defect and its consequences for daily life and the future.**  **Knows when and where they should seek medical care.**  **Can tell others about their heart defect.** |  | |  |

| Person centred transfer plan – Visit 1 Resources/obstacles – Motivation – Personal goals |
| --- |
| **Goal setting (develop independence and selfcare ability)** |
|  |
|  |
|  |
|  |
|  |
| **What should I do and how? When?** |
|  |
|  |
|  |
|  |
|  |
|  |
| **Personal resources and capacity (how can they be used to reach the set goals?)** |
|  |
|  |
|  |
|  |
|  |
|  |
|  |
| **In need of support (What do I need help with?):** |
|  |
|  |
|  |
|  |
|  |
|  |

**VISIT 2**

| **Preparations for Visit 2** | |
| --- | --- |
| Update via medical journal |  |
| Send appointment request |  |
| Date and time agreed |  |
| Prepared documentation |  |
| Book meeting room |  |
| Ensure the material to be used is there (LAS scale, paper, pens, computer, plastic heart model etc) |  |
| Send text message reminder about visit to adolescent |  |
| **To check with the paediatric cardiologist in charge**  **– important aspects in relation to the person and the heart defect** | |
| Health status |  |
| Physical activity |  |
| Medication |  |
| Pregnancy |  |
| Contraceptives |  |
| Prognosis (short and long term) |  |
| Future need of intervention |  |
| Future need of medical checks and at what level of care |  |
| Advice regarding choice of education and profession |  |
| Advice about travel (to think about) |  |
| Driving licence |  |
| Endocarditis prophylaxis |  |
| Tattoos and piercings |  |

| Patient reported health status/skills and resources – Visit 2 | | |  |
| --- | --- | --- | --- |
| **Date** | |  | |
| Health (1-10) LAS | |  | |
| LAS (1-10)  Based on person’s needs  Specify your assessment | |  | |
| LAS (1-10)  Based on person’s needs  Specify your assessment | |  | |
| **HEADSS – Visit 2** | | |  |
| **H**  **Home** |  | |  |
| **E**  **Education**  **Employment** |  | |  |
| **A**  **Activities** |  | |  |
| **D Drugs/alcohol/**  **smoking** |  | |  |
| **D**  **Depression**  **Mental health** |  | |  |
| **S**  **Sexuality** |  | |  |
| **Strengths** |  | |  |
| **Participation** |  | |  |
| **Knows about their heart defect and its consequences for daily life and the future.**  **Knows when and where they should seek medical care**  **Can tell others about their heart defect.** |  | |  |

| Person centred Transfer plan - Visit 2 Resources/obstacles – Motivation – Personal goals |
| --- |
| **Goal setting (develop independence and selfcare ability)** |
|  |
|  |
|  |
|  |
|  |
| **What should I do and how? When?** |
|  |
|  |
|  |
|  |
|  |
|  |
| **Personal resources and capacity (how can they be used to reach the set goals?)** |
|  |
|  |
|  |
|  |
|  |
|  |
|  |
| **In need of support (What do I need help with?):** |
|  |
|  |
|  |
|  |
|  |
|  |

| **Preparations for the group activity day** | | |
| --- | --- | --- |
| Written invitation to the group activity day sent to the adolescent and parent one month in advance |  |  |
| RSVP received (yes/no) |  |  |
| If no, reminder sent after two weeks (in writing and by text message) |  |  |

**VISIT 3**

| **Preparations for Visit 3** | |
| --- | --- |
| Send appointment request |  |
| Date and time agreed |  |
| Prepared documentation |  |
| Book meeting room |  |
| Ensure the material to be used is there (LAS scale, paper, pens, computer, plastic heart model etc) |  |
| Update via medical journal |  |
| Send text message reminder about visit to the adolescent |  |
| **To check with the paediatric cardiologist in charge prior to transfer – important aspects in relation to the person and the heart defect** | |
| Health status |  |
| Physical activity |  |
| Medication |  |
| Pregnancy |  |
| Contraceptives |  |
| Prognosis (short and long term) |  |
| Future need of intervention |  |
| Future need of medical checks and at what level of care |  |
| Advice regarding choice of education and profession |  |
| Advice about travel (to think about) |  |
| Driving licence |  |
| Endocarditis prophylaxis |  |
| Tattoos and piercings |  |
| **Planning for meeting with GUCH** | |
| Agreed time with GUCH-nurse/doctor |  |
| Medical epicrisis sent |  |
| Personal narrative sent |  |

| **Patient reported health status/skills and resources – Visit 3** | | |  |
| --- | --- | --- | --- |
| **Date** | |  | |
| Health (1-10) LAS | |  | |
| LAS (1-10)  Based on person’s needs  Specify your assessment | |  | |
| LAS (1-10)  Based on person’s needs  Specify your assessment | |  | |
| **HEADSS – Visit 3** | | |  |
| **H**  **Home** |  | |  |
| **E**  **Education**  **Employment** |  | |  |
| **A**  **Activities** |  | |  |
| **D Drugs/alcohol/**  **smoking** |  | |  |
| **D**  **Depression**  **Mental health** |  | |  |
| **S**  **Sexuality** |  | |  |
| **Strengths** |  | |  |
| **Participation** |  | |  |
| **Knows about their heart defect and its consequences for daily life and the future.**  **Knows when and where they should seek medical care**  **Can tell others about their heart defect.** |  | |  |

| Person centred Transfer plan – Visit 3 Resources/obstacles – Motivation – Personal goals |
| --- |
| **Goal setting (develop independence and selfcare ability)** |
|  |
|  |
|  |
|  |
|  |
| **What should I do and how? When?** |
|  |
|  |
|  |
|  |
|  |
|  |
| **Personal resources and capacity (how can they be used to reach the set goals?)** |
|  |
|  |
|  |
|  |
|  |
|  |
|  |
| **In need of support (What do I need help with?):** |
|  |
|  |
|  |
|  |
|  |
|  |

## EVALUATION

## PERSON CENTRED TRANSFER PLAN FOR ADOLESCENTS WITH CONGENITAL HEART DEFECTS

| **Evaluation (C)** | | |
| --- | --- | --- |
| The patient thinks the transfer plan has been adhered to | Yes | No |
| The patient thinks the transfer has been planned in a satisfactory manner | Yes | No |
| The parents think the transfer has been planned in a satisfactory manner | Yes | No |
