# Supplementary material for: Implementation fidelity of a transition program for adolescents with congenital heart disease: the STEPSTONES project
Source: BMC Health Serv Res. 2022 Feb 5;22:153. doi: 10.1186/s12913-022-07549-7 (PMC8817652; doi:10.1186/s12913-022-07549-7)
Supplement: Supplementary file 3 — Additional file 3. [file 12913_2022_7549_MOESM3_ESM.docx]

**Additional file 3 – Interview guides**

**Interview guides for paired interviews with transition coordinators**

*Interview 1*

1. Can you tell me about your experience of the first meeting with the youths?
2. Can you describe how you structure the meeting?
3. Can you tell me what you think about using the materials/tools in the discussion/meeting with the youth? (potentially probing questions)
4. What kind of preparations are made before the discussions?
5. Can you tell me about any meetings that particularly affected you?
6. Can you describe the parents’ participation in the meetings?
7. What parts of the meetings are more difficult to perform?
8. What parts of the meetings are easier to perform?
9. Is there anything associated with the meetings that is difficult to perform?
10. What is your experience of the administrative work involved with the program? (that is not related to the study)
11. Can you reflect on what it’s like to collaborate with the other healthcare staff in connection with the meetings with the youths?
12. What is your experience of support for your work from others in your environment? Can you tell me more about what aspects of this support are good/less good?
13. How does the space work for meeting with the youths?

*Interview 2*

**Second discussion**

1. How do you structure it? How do you prepare?
2. How does the conversation compare to the first one?
3. In terms of content, what is different?
4. How is the formulation of goals working out?

**Mild/moderate heart defects not troubled by symptoms**

1. How are the patients doing now?
2. Is it difficult? What and why is it difficult?

**Transfer**

1. What are the youths’/parents’ thoughts on this?
2. How do you handle this?

**Psychosocially heavy subjects**

1. What’s the situation now? How often does this happen?
2. How do you handle this now?
3. What kind of support can you access when you have to deal with these issues?

**Collaboration**

1. How do doctors, nurses, parents or other involved parties collaborate?
2. Are more professions needed around the transition team?

**Transition coordinator role**

1. How has your role changed/developed over time? What new things do you do now with the youths?
2. How do you see your role? (from the youths’, parents’ and team’s perspective)
3. What do you think of your knowledge in relation to managing your work? Is more knowledge needed – if so, what?

*Interview 3*

**Third discussion/visit**

1. Can you tell me your experience of the third visit as compared to the first and second?
2. How do you structure it? How do you prepare?
3. In terms of content, what is different?
4. How is the transfer of goals working? And cooperation with the nurse at ACHD?

**Last time we talked a bit about mild/moderate heart defects not troubled by symptoms**

1. How are those patients doing now? How are you working with them on the program?
2. Is it difficult? What and why is it difficult?

**Last time we also talked about psychosocially heavy subjects**

1. What’s the situation now? How has it changed?
2. How do you handle this now?
3. What kind of support can you access when you have to deal with these issues?

**Information evening for youths and parents**

1. What did people think of it?
2. What was difficult/easy/unexpected
3. Improvements – what would you have liked to have seen?

**The discussions in general**

1. What is the most difficult thing to do in the meeting?
2. What works/doesn’t work with the youths?
3. What do the youths/parents think of the program/content? Have you received feedback?

**The program in general**

1. What is your view of the program as a whole? Is there anything missing? Improvements?
2. What should have been done differently in the program? (Future implementation?)

*Interview 4*

1. How do you think your work as transition coordinator **and the execution of the program in general** is going?

- How has this changed over time?
- What has changed?
- Has anything got more difficult/easier? What? Why?
- Collaboration with others (e.g. doctors, ACHD)?
- What are the meetings with the youths like?
- What works/doesn’t work in the meeting?
- Have you received any feedback from the youths/parents about the meetings/study? What do they say?
- What is hard to perform in the meeting?
- How is HEADDS working? What possibilities/difficulties are there?
- How are the various apps being used now? (Concept map, shared decision-making etc.) Do you use it as much as you did at the beginning? How does your usage of these differ as the meetings progress?

1. How is the **documentation** working?

- What is difficult?
- How much time do you think it takes? What is it that takes time?

1. How is the **goal-setting** going?

- This has been difficult before – how are you overcoming this?
- What strategies have you found for helping the youths formulate goals?
- Have you got examples of situations when it didn’t work at all or when it worked really well?

1. How do you think **ordinary care** is affected by the intervention? (both pediatric and adult care)

- In what way does the intervention affect it?
- Are you formulating goals that involve ordinary care?

1. Can you describe how the recruitment process was carried out and developed?
   - What information about the study did you give to the youths and parents at recruitment?
   - What information channels did you use to recruit them? Please describe.
   - What questions have the parents/youths asked about the study?
   - What kind of feedback have the youths/parents given regarding the information?
   - How do you explain research concepts such as randomization, consent, voluntary to the youths and parents? How do you think they have absorbed this?
