# Supplementary material for: Implementation fidelity of a transition program for adolescents with congenital heart disease: the STEPSTONES project
Source: BMC Health Serv Res. 2022 Feb 5;22:153. doi: 10.1186/s12913-022-07549-7 (PMC8817652; doi:10.1186/s12913-022-07549-7)
Supplement: Supplementary file 4 — Additional file 4. [file 12913_2022_7549_MOESM4_ESM.docx]

**Interview guide focus group with ACHD nurses**

1. I’d like to start by asking you what your impression of the study was when you were informed it was going to start?
2. The part of the study your participation has mainly concerned is the transition visit with the transition coordinator and the young adult. Can you describe to me your experience of those visits?
3. In what way did the transition visits you attended in the study compare with an ordinary visit when you meet a young person who has just been transferred from pediatric care? Or at a transition meeting in ordinary routine care?
4. How was the collaboration with the transition coordinator before and during the visit?
5. Did you feel that the young people who participated in the study and transitioned were more prepared before transition compared to people who did not participate?
6. Some of you participated in an information evening for youths and parents in the study where we gathered staff from adult health care and had discussions all together. Can you tell me a little about what it was like to participate in that?
7. Part of the transition program is to work with the youth’s personal narrative to set goals for their health and wellbeing. We are also trying to identify what personal resources they have to achieve these goals and work with them to document this in the transition plan. How do you envisage the opportunity to carry on working this way with young adults in adult care?
8. If this transition program is effective, the aim for the future is to implement it as part of ordinary care. What possibilities for this do you envisage?
9. And in contrast, what obstacles to future implementation do you envisage?
10. Finally, do you think a transition program such as this can help youths with congenital heart defects prepare better for transition and for adult life with a chronic disease, and if so, in what way?
11. I have asked all my questions. Thank you for agreeing to participate. Is there anything more you’d like to add or highlight?
